# Review of behaviour change interventions to reduce population salt intake

**DOI:** 10.1186/s12966-017-0467-1

**Published:** 2017-02-08

**Authors:** Kathy Trieu, Emma McMahon, Joseph Alvin Santos, Adrian Bauman, Kellie-Ann Jolly, Bruce Bolam, Jacqui Webster

**Affiliations:** 1 The George Institute for Global Health, The University of Sydney, PO Box M20, Missenden Rd, Camperdown, NSW 2050 Australia; 2 Menzies School of Health Research, Royal Hospital Campus, Rocklands Dr, Tiwi, NT 0810 Australia; 3 Prevention Research Collaboration, School of Public Health, Charles Perkins Centre (D17), The University of Sydney, Camperdown, NSW 2006 Australia; 4 National Heart Foundation (Victorian Division), 12/500 Collins St, Melbourne, VIC 3000 Australia; 5 Victorian Health Promotion Foundation, 15-31 Pelham St, Carlton, VIC 3053 Australia

**Keywords:** Sodium, Salt, Nutrition, Public health, Dietary intervention, Hypertension, Cardiovascular disease

## Abstract

**Background:**

Excess salt intake is a major cause of raised blood pressure—the leading risk factor for death and disability worldwide. Although behaviour change interventions such as awareness campaigns and health education programs are implemented to reduce salt intake, their effectiveness is unclear. This global systematic review investigates the impact of population-level behaviour change interventions that aim to reduce salt intake.

**Methods:**

A search for published and grey literature was conducted using PubMed, Cochrane Library, Embase, Web of Science, Sage, Scopus, OpenGrey, Google Scholar and other relevant organizations’ websites. Studies were included if 1) published between 2005 and 2015; 2) the education or awareness-raising interventions were aimed at the population or sub-population and 3) salt intake and/or salt-related behaviours were outcome measures. Study and intervention characteristics were extracted for the descriptive synthesis and study quality was assessed.

**Results:**

Twenty two studies involving 41,448 participants were included. Most were conducted in high income countries (*n* = 16), targeting adults (*n* = 21) in the general population (*n* = 16). Behaviour change interventions were categorised as health education interventions (*n* = 14), public awareness campaigns (*n* = 4) and multi-component interventions (including both health education and awareness campaigns, *n* = 4). 19 of the 22 studies demonstrated significant reductions in estimated salt intake and/or improvement in salt-related behaviours. All studies showed high risk of bias in one or more domains. Of the 10 higher quality studies, 5 found a significant effect on salt intake or salt behaviours based on the more objective outcome assessment method.

**Conclusion:**

Based on moderate quality of evidence, population-level behaviour change interventions can improve salt-related behaviours and/or reduce salt intake. However, closer analysis of higher quality studies show inconsistent evidence of the effectiveness and limited effect sizes suggest the implementation of education and awareness-raising interventions alone are unlikely to be adequate in reducing population salt intake to the recommended levels. A framework which guides rigorous research and evaluation of population-level interventions in real-world settings would help understand and support more effective implementation of interventions to reduce salt intake.

## Background

Globally each year, 1.65 million cardiovascular-related deaths are caused by people consuming too much salt [1]. Excess salt intake is a major contributor to high blood pressure, the leading individual risk factor for death and disease burden worldwide [2]. There is strong evidence for the efficacy of reducing salt intake to lower blood pressure (BP) [3] and the subsequent impact on cardiovascular disease [4]. Population salt reduction has been identified as one of the top five priority interventions to prevent non-communicable diseases (NCDs) based on parameters such as health effects, cost effectiveness, low implementation costs and political and financial feasibility [5]. In 2013, reducing salt intake by 30% was one of nine global targets endorsed by all World Health Organization (WHO) Member States to reduce NCDs [6]. Increasingly countries are developing interventions in response to this, with a recent review identifying 75 countries with national salt reduction strategies in 2014, double that reported in 2010 [7]. In 2014, 12 countries (10 high income and 2 upper-middle income countries) have reported reductions in population salt intake and several community trials including those in Portugal and China have demonstrated reductions in salt intake and BP [7–10]. However, most programs are multi-faceted and there is still uncertainty about the specific initiatives or elements of the strategy that are central to their success.

The WHO has classified national-level salt reduction interventions into three main pillars: consumer behaviour, product reformulation and environmental changes [11]. Common to nearly all national salt reduction programs are educational or awareness-raising interventions that provide consumers with information, education or skills to reduce salt consumption [7]. These interventions aim to change people’s salt-related behaviour through strengthening knowledge on salt and its adverse effects and skills to help lower salt intake. Such programs can potentially have a larger impact in those low- and middle- income countries (LMICs) where the primary source of salt is discretionary salt added by the individual during cooking or at the table [12]. In countries where the majority of salt comes from processed or packaged foods, these educational behaviour change programs can also help support product reformulation and environmental changes by generating demand for lower-salt products in the market. However, because behaviour change strategies are usually implemented as part of multi-faceted programs, most evaluations to date have not attempted to differentiate the impact of the intervention components.

Previous reviews have focussed on behaviour change programs that target individuals, such as one-to-one nutrition counselling, and found such interventions to be highly intensive for individuals and unsustainable as a population-wide strategy [13]. The objective of this review is to investigate the characteristics and effectiveness of more recent behaviour change programs which aim to lower salt intake in whole populations or sub-populations (e.g. people in schools or those at increased risk). This will inform future guidance on the implementation of salt reduction strategies including which consumer education and awareness interventions are most likely to be effective in reducing population salt intake.

## Methods

### Literature search

A search strategy was developed to identify studies with behaviour change interventions to reduce salt intake published in English between February 2005 to February 2015 in PubMed, Cochrane Library, Embase, Web of Science, Sage and Scopus. The search was limited to studies published in the last 10 years to ensure contemporary evidence of effective interventions was captured and the review findings were applicable to the current environment. The search strategy included three categories of search terms: salt terms (salt, sodium, dietary salt, dietary sodium), outcome terms (reduce, decrease, change, lower, alter) and intervention terms (intervention, education, promotion, social marketing, media, campaign, program, initiative, strategy, project, activity). In addition, similar search terms were used to identify potentially relevant grey literature from OpenGrey, Google Scholar, World Health Organization and regional office websites, governmental websites, scientific or non-governmental organization websites.

### Inclusion/exclusion criteria

Two authors (KT and EM) independently screened the abstracts and full text for studies that evaluated a behaviour change intervention to reduce salt intake at a population-level using the below specified criteria. Any discrepancies in selections were discussed until a consensus was reached.

#### Types of studies/participants

All human studies were included such as randomised controlled trials (RCTs), controlled and uncontrolled before-after intervention studies and serial cross sectional surveys conducted before and after interventions from both peer reviewed journals and the grey literature (unpublished reports). Studies of all populations, of any age and living in any region worldwide were included in the review as were studies of population subgroups.

#### Types of interventions

This review focused on consumer education and awareness raising programs that aimed to improve peoples’ behaviour related to salt intake at a population or sub-population level (an organizational e.g. workplace or schools, community, state or national level). Studies of interventions that provided individualised nutrition counselling or advice were excluded as these have been reviewed elsewhere [13]. Studies where the impact of the behaviour change intervention could not be distinguished from other programs such as food reformulation, healthy food procurement or fiscal policies were excluded from the review.

#### Outcome measures

Studies that reported outcomes related to change in salt consumption behaviour assessed through a) estimated salt intake or b) salt-related behaviours using any method were included. Studies were excluded if the outcome measures were limited to change in knowledge or attitudes related to salt.

### Data extraction and analysis

Two review authors (KT and JS) collaboratively extracted data using modified data extraction forms designed for this review. Information regarding study design, intervention approach, participants, outcome measures, statistical analysis and results was collected. Intervention effectiveness was determined by the difference in salt intake or behaviour, between the intervention and control group, or over time. Two independent authors (KT and JS) assessed the quality of studies using the Cochrane Collaboration risk of bias tool for RCTs and a modified tool for non-RCTs. The tool assessed each study for selection bias, measurement bias, attrition bias, reporting bias and bias related to exposure to other factors than the salt reduction intervention [14]. Disagreements were resolved through discussion. Data synthesis was based on all included studies. Significant heterogeneity meant a meta-analysis could not be undertaken.

## Results

### Search results

**Fig. 1:**
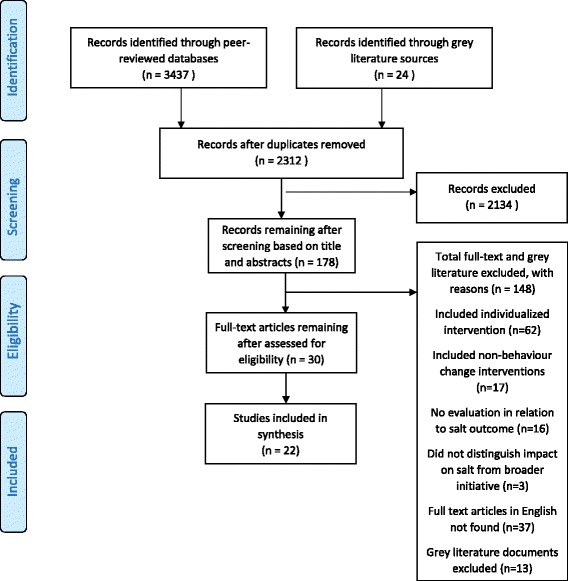
PRISMA flow chart of included studies of salt reduction behaviour change interventions

The peer-reviewed literature search identified 3437 records with 2288 remaining after removing duplicates. Titles and abstracts were screened leaving 154 full text articles to be assessed for eligibility. Of these, 37 full text articles could not be found or were unavailable in English and 98 full-texts were excluded. The reasons for excluding these studies are: 62 studies had interventions which included individualised behaviour change activities, 17 studies had interventions that were not solely behaviour-change interventions (and the impact of the behaviour change intervention could not be distinguished), 16 studies did not evaluate the effectiveness of interventions in relation to salt intake or behaviour and 3 studies did not distinguish the intervention’s impact on salt behaviour from the impact of the broader initiative. The remaining 19 peer reviewed articles, reporting 16 studies met the criteria. Of 24 documents retrieved from the grey literature search, 11 documents reporting 6 studies met the eligibility criteria. A total of 30 papers, reporting 22 studies were included in the descriptive synthesis (Fig. 1).

### Description of studies

A total of 41,448 participants were represented in the 22 studies. Sample sizes ranged from 21 to 30,799 participants with a median pre-intervention sample size of 188 (inter-quartile range of 64–801). There were four studies each in China [15–18] and UK [19–22], three in the United States [23–25], two each in Australia [26, 27], Japan [28, 29] and Portugal [30, 31] and one each in Canada [32], Ghana [33], Iran [34], Ireland [35] and Italy [36]. Six studies were undertaken in LMICs and the remainder were in high income countries. Sixteen studies were delivered to the general population and the other six studies were in high risk groups such as patients with hypertension, stroke or heart failure (Table 1).

**Table 1:** Characteristics of included studies and results

First author (Year) Country	Study design	Participants	Intervention description	Study duration	Study findings
Health education					
Ferrara (2012) [36] Italy	RCT	Risk group—hypertensive patients from an outpatient clinic (I: 94, C: 94)	Patients were educated by doctors and dietitians on the link between salt intake and hypertension, importance of salt reduction and how to reduce salt intake. Delivery setting: clinic	1 year	Overall, significant net reduction in salt intake by 1.85 g/d (*p* < 0.001). I: reduction in salt intake from 7.18 g/d (SD 2.42 g/d) to 5.06 g/d (SD 1.75 g/d) after 1 year (*p* < 0.001). C: reduction in salt intake from 6.58 g/d (SD 2.26 g/d) to 6.31 g/d (SD 2.03 g/d) (*p* = 0.03).
Cotter (2013) [31] Portugal	RCT (2 intervention groups, 1 control)	General population—school children in their 5th and 6th years of education (I_Practical: 53, I_Theory: 43, C: 31)	Practical group: lecture on the potential dangers of salt intake and school gardening to grow herbs as substitutes of salt in food preparation. Theory group: lectures on the danger of salt intake. Delivery setting: school	6 months	No significant differences between the 3 groups (practical, theory and control). I_Practical: significant reduction in salt intake by 1.1 g/d (SD 2.5 g/d) pre post intervention. I_Theory: non-significant reduction in salt intake by 0.6 g/d (SD 3.2 g/d). C: non-significant reduction in salt intake by 0.4 g/d (SD 2.4 g/d).
Veroff (2012) [23] United States	RCT	Risk group—adults with heart failure (I: 246, C: 234)	Participants were mailed education materials and medical decision aid (evidence-based DVD and booklet) to help manage heart failure. Delivery setting: community	4 weeks	No significant difference in the percentage of participants that reported following a low-sodium diet every day or most days between groups. I: 83%. C: 77%.
Cappuccio (2006) [33] Ghana	Community cluster RCT	General population—aged 40–75 (I: 399, C: 402)	Sessions led by community health workers on not adding salt to food and in cooking, salty foods to limit, low salt cooking practices (open to all in community). Delivery setting: community	6 months	No significant change in salt intake between intervention and control group. I: reduction in salt intake by 0.52 g/d from baseline to follow up. C: reduction in salt intake by 0.84 g/d.
Lin (2013) [24], Svetkey (2009) [56] United States	Nested 2x2 RCT [Only included patient intervention]	Risk group—hypertensive patients (I: 256, C: 269)	Behavioural interventionists delivered education to reduce salt intake through promoting self-monitoring, goal setting and motivational interviewing techniques. Delivery setting: clinic	18 months	No significant difference in 24- h urinary sodium excretion between groups at 6 months. Salt intake based on FFQ showed that intervention patients significantly decreased intakes compared to controls (*p* < 0.05).
Chen (2008) [15] China	Non-randomised CT	General population—110,000 workplace employees	Education and promotion on reducing salt intake and health professional training. Delivery setting: workplace	6 years	Significant net reduction of 3.9 g/d (*p* < 0.05). I: reduction in salt intake from 16 to 10.6 g/d from baseline in 1987 to follow up in 1995. C: reduction in salt intake from 16.9 to 15.5 g/d.
Kitaoka (2013) [28] Japan	Non-randomised CT	Risk group—men aged 40–75 years with SBP of 130–180 mmHg and DBP of 85–110 mmHg (I: 38, C: 26)	Dietitian led lectures on diet for reducing BP, self-monitoring and cooking instructions on how to prepare low salt meals and measure seasoning. Delivery setting: community	5 months	Based on spot urine, I: salt intake decreased from 12.3 g/d at baseline to 10.6 g/d at follow-up (*p* = 0.025). C: salt intake decreased from 15.5 g/d to 13.3 g/d (*p* = 0.014). Based on FFQ, intervention patients significantly reduced consumption of preserved vegetables compared to control (*p* = 0.039). Based on dietary habits questionnaire, intervention group significantly reduced consumption of noodle soup compared to control.
Ireland (2010) [26] Australia	Parallel group RT (2 treatment groups)	Population—healthy adults (I_FSANZ: 21 I_Tick: 22)	Nutritionist educated groups to choose low-salt foods using the Tick symbol or the FSANZ guideline of <120 mg/100 g sodium. Both groups were also provided with a list of low-sodium foods. Delivery setting: community	8 weeks	I_FSANZ: significant reduction in salt intake by 1.99 g/d based on 24- h urine at week 8 (*p* < 0.05). Also significant reduction by 3.27 g/d of salt based on multiple 24- h diet recall (*p* = 0.003). I_Tick: significant reduction in salt intake based on 24-h urine by 0.88 g/d at week 8 (*p* < 0.05). Non-significant reduction in salt intake based on multiple 24 h diet.
Lu (2015) [17] China	Parallel group RT (3 treatment groups)	Risk group—hypertensive patients (Group 1: 116, Group 2: 114, Group 3: 117)	Group 1: reading materials for self-learning. Group 2: lectures on hypertension. Group 3: interactive workshop using visual health education tools such as animation, food models, salt spoons, and CVD models. Delivery setting: community	2 years	Based on monthly salt weighing, all three groups demonstrated significant increases in the number of people with salt intake <6 g/d between baseline and post-intervention, but it was progressively greater from group 1 (self-learning) to group 2 (regular lecture) and group 3 (interactive education).
Resnick (2014) [25] USA	Before and after study	Population—29 low income residents living in the senior housing facility	Nurses led lectures on heart healthy diets and medication adherence. Remaining 11 weeks included provision of health tips to avoid high salt foods led by a lay trainer. Delivery setting: community	3 months	Significant reduction in mean salt intake from 13.49 g/d (SE 1.58) at baseline to 9.08 g/d (SE 1.30) post intervention (*p* = 0.01) based on FFQs.
White (2013) [27] Australia	Before and after pilot study (Mixed methods)	Risk group—21 patients with diagnosis of stroke in community	A multi-professional community based team led education session on various topics such as stroke risk factors, nutrition and diet. Delivery setting: community	1 year	Statistically significant reduction in salt intake from 10.62 g/d (SD 2.62 g/d) in baseline to 8.62 g/d (SD 2.36 g/d) in the post program based on questionnaires (*p* = 0.01). At 3 months follow-up, mean salt intake of 8.6 g/d (SD 1.68 g/d) was sustained.
Bogle (2008) [19] UK	Before and after study	Population—African-Caribbean, Black African, Asian, Irish and Turkish ethnic groups (Shop tours: 23; Cook and eat sessions: 37)	“Cook and Eat” programmes and grocery shopping tours were delivered by dietitians/nutritionists to provide advice on risks of high salt intake, cooking low-salt meals and low-salt practices interpreting food labels. Delivery setting: community	4 weeks	Self-reported behaviour about adding salt in cooking significantly decreased (*p* = 0.04). Significant increase in participants checking sodium label of foods when shopping. At 3 months post intervention, 65% reported reducing salt by ‘a lot’ in their diet and 21.6% ‘somewhat’ reduced salt intake.
Chen (2013) [16] China	RCT	Population—adults responsible for home cooking (I: 141, C: 107)	Education about the amount of salt that should be eaten, provision of a salt-restriction spoon and how to correctly use the salt restriction spoon. Delivery setting: community	7 months	No significant difference in salt intake between intervention and control group based on 24-h urine. Based on salt weighing after 6 months, there was a significant difference between groups by time, with 1.42 g/d reduction in intervention compared to 0.28 g/d in control group (*p* = 0.041).
Fujii (2009) [29] Japan	Before and after study	Population—185 workplace employees	Participants were provided lifestyle advice (including advice to reduce salt) through a computer-based lifestyle modification support tool. Delivery setting: workplace	4 months	Significant increase in the proportion of women practicing low salt intake behaviours post intervention (63.3 to 75%, *p* = 0.039). No significant increase in men practicing low salt intake behaviours.
Public awareness campaigns					
Papadakis (2010) [32] Canada	Before and after controlled trial	Population—adults aged 35–50 living in Champlain District of Ontario (I: 1565, C: 1565)	Bilingual mass media campaign to reduce consumption of high sodium processed foods delivered through TV, radio, print and web advertisements, and 100 editorial stories. Delivery setting: community	2 years	At 6 months there was a significant reduction in salt added to foods in the intervention community compared to control. Intervention participants at follow-up were 1.27 (95% CI:1.09,1.49) times more likely to check nutrition labels for sodium content in foods compared to baseline.
Wyness (2012) [20], Millett (2012) [38], He (2014) [54] UK	Serial cross sectional survey	Population—United Kingdom (2000 nationally representative adults)	Mass media campaign involved TV, radio, press, poster ads, leaflets and other materials to raise awareness, highlight 6 g/d target and provide tips to reduce salt. Delivery setting: national	6 years	Increase in proportion of adults self-reporting efforts to reduce salt in their diet from 34% in 2004 to 43% in 2009. Increase in adults checking food labels to find salt content from 29% in 2004 to 50% in 2009.
Safefood (2006) [35] Ireland	Before and after study	Population—adults aged 15–74 years in Ireland (Billboard: 172, radio ad: 235)	‘Already Salted’ campaign on billboard and radio advertisement to raise awareness about level of salt in foods and provide tips on how to reduce salt intake. Delivery setting: national	6 weeks	Amongst adults who saw the billboard campaign, there was an increase in the proportion reporting that they had changed their salt behaviour (to 37%) from 2003 to 2006. Amongst adults who heard the radio ad, there was an increase in the proportion reporting that they had changed salt behaviour (to 25%) from 2003 to 2005.
Martins (2009) [30] Portugal	Before and after study	Population—Portugal	Public education on the harmful consequences of salt and hypertension using mass media (TV, newspapers, radio). Delivery setting: national	-	Increase in the proportion of adults who reported that they had changed their salt intake (to 44%) and substantially reduced their salt intake (to 25%) from 2007.
Multi-component education					
Zhang (2014) [18] China	Cross sectional study (with intervention vs control counties)	Population—nationally representative sample of residents in provinces and counties in China (I: 17684, C: 13115)	Public awareness campaigns and multisector approach involving health professionals, employers, schools and community organizations to distribute health educational messages through a wide range of channels. Delivery setting: community	8 years	People living in the intervention counties were 2.16 (95%CI 1.50, 3.12) (unadjusted)/1.98 (95% CI 1.41, 2.76) (adjusted) times more likely to report having reduced salt consumption than people in the control counties.
Khosravi (2012) [34] Iran	Serial cross sectional survey	Population—representative sample of normotensive Iranian adults (Before: 374, After: 806)	Communities received education through mass media and face-to-face education programs by health workers, and educational materials provided in schools and workplaces. Delivery setting: community	6 years	Statistically significant reduction in salt intake from 12.52 g/d in 2001–02 to 10.66 g/d in 2007 (*p* < 0.01) based on 24 h urine collection.
Drummond (2008) [21] UK	Before and after study	Population—South Asian and Caribbean communities (Before: 100, After: 66)	Distribution of materials to the public including flyers, leaflets and booklet in combination with education sessions on how to read salt content in labels and how it relates to max daily amount, effects of salt on health, practical ways to reduce salt and cook and taste sessions. Delivery setting: community	1 year	Between baseline and post intervention, there was an increase in the proportion of people who claimed to ‘always’ check the label for salt content of food products when shopping from 12 to 28% and to ‘always’ choose lower salt options when buying food from 23 to 43%. There was also a decrease in those who claimed to ‘always’ add salt to food at the table from 11 to 5%.
Surpluss (2008) [22] UK	Before and after study	Population—workplace employees, men on low income and of an ethnic background (Question 1: Before: 149, After: 91) (Question 2: Before: 272, After: 136)	Distribution of posters, flyers, postcards, ads in the workplace newspapers, information tools for dining tables and health education, healthy cooking and low-salt tasting sessions delivered by experts. Delivery setting: workplace	1 month	Between baseline and post intervention, there was a reduction in the proportion of men who reported ‘always’ adding salt to food when cooking from 48 to 23% (Question 1) and an increase in the proportion of men who reported checking food labels for salt content prior to purchase or eating from 11 to 26% (Question 2).

*RCT* randomized controlled trial, *CT* controlled trials, *RT* randomized trial, *I* intervention group, *C* control group, *FFQ* food frequency questionnaire, *FSANZ* Food Standards Australia New Zealand sodium guideline, *Tick* Heart Foundation Tick Scheme, *CVD* cardiovascular disease

### Quality of studies

**Fig. 2:**
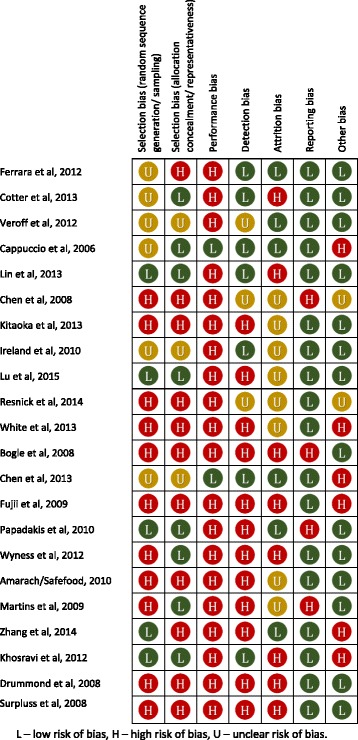
Summary of risk of bias assessment of studies adapted from Cochrane

All studies had one or more domains characterised as high risk of bias (Fig. 2). Ten studies had better quality, based on having more low risk than high risk domains out of a total 7 domains [16, 17, 23, 24, 26, 31–34, 36]. The remaining 12 studies were of low quality [15, 18–22, 25, 27–30, 35]. All except two studies had high risk of performance bias due to lack of blinding or potential confounding [16, 33]. The 12 low quality studies were also rated as high risk in relation to selection bias, due to a non-random selection of participants and detection bias due to subjective and unreliable outcome measures such as self-reported salt behaviours [15, 18–22, 25, 27–30, 35].

### Types of interventions

Half of the studies had interventions specific to reducing salt intake and the other half were part of broader interventions to prevent or manage hypertension or CVD. Three interventions were delivered nation-wide (in the UK [20], Ireland [37] and Portugal [30]), 13 were delivered in the community setting, three were in workplaces [15, 22, 29], two in health clinics [24, 36] and one in a school, targeting children [31]. Five studies specified that the interventions were based on behaviour change models or theories [24, 25, 27, 29, 32].

The interventions were categorised into (Table 1):

1. Health education characterised by the provision of in-depth information about salt reduction delivered directly to groups of people;
2. Public awareness campaigns to change behaviour on a large scale, often characterised by short messages delivered through mass media, print and digital media; and
3. Multi-component education interventions which included both health education and awareness campaigns.

#### Health education

Fourteen studies evaluated 18 health education interventions. All except three were led by an educator; eight were delivered by health professionals (dietitians, doctors, nurses or community health workers) [19, 24–28, 33, 36], one intervention was delivered by behaviour change interventionists [24] and the remaining studies did not describe who delivered the education. Of the three interventions not led by an educator, two involved print-materials and a DVD mailed for self-education [17, 23] and the other involved a computer-based lifestyle modification support tool which provided participants with lifestyle advice and encouraged goal-setting [29].

Most health education interventions were delivered through didactic group lectures. Eight also included interactive or practical approaches such as low-salt cooking demonstrations and grocery shop tours [19, 28], workshops using food and disease models and animation [17], gardening of herbs that could be used as alternatives to salt [31], salt-restriction-spoons [16], specific education on two types of nutrition labels [26] and a computer-based lifestyle modification support program [29]. In-depth health education interventions were used in all six studies that targeted high risk populations [17, 23, 24, 27, 28, 36].

#### Public Awareness Campaigns

Four studies evaluated public awareness campaigns, three delivered nation-wide and one in a community. Program duration ranged from 6 weeks to 5 years. The campaigns in the United Kingdom [20, 38], Champlain District of Ontario [32], Ireland [35, 37] and Portugal [39] all used multiple modes of media such as television, radio, web and print media. The UK’s Food Standard Agency (FSA) campaign featured 4 phases with key messages on the risks of high salt intake on health, the 6 g/d salt target, sources of salt and advice to check labels for salt content over the 5 years [20, 38]. Similarly the Champlain District of Ontario ‘Give your head a shake’ sodium reduction campaign provided practical tips to reduce salt intake and highlighted foods high in salt [32]. In Ireland and Portugal the campaign messages were more focused on raising awareness about the adverse health risks of excess salt consumption [35, 37, 39].

#### Multi-component behaviour change programs

Four studies included both health education and awareness campaigns, three delivered in the community and one in a workplace [18, 21, 22, 34]. The awareness campaigns used various delivery modes, such as printed brochures, posters, billboards and mass media, in conjunction with in-depth education delivered to communities, school or organization groups. All four interventions targeted ethnic population groups (Asian, Iranian and Caribbean) who are more likely to add discretionary salt or salty condiments during cooking or at the table. Intervention duration ranged from 1 month to 8 years [18, 21, 22, 34].

### Effects of interventions

Half of the studies (*n* = 12) measured outcomes based on dietary salt intake and the other half based on self-reported behaviours with 2 studies measuring both. Salt intake was estimated from 24 h urinary sodium excretion (gold standard) in 6 studies [16, 24, 26, 31, 33, 34], spot urine samples in 1 study [28], dietary surveys in 7 [15, 24–27, 36] and salt weighing in 2 studies [16, 17] (4 studies used more than one method). The other 12 studies measured the intervention’s impact using questionnaires about salt-lowering behaviours such as adding less salt at the table or during cooking and checking salt content on food labels [16, 18–23, 28–30, 32, 35].

Overall, 19 of 22 studies found the behavioural interventions had a statistically significant result based on either decreases in estimated daily salt intake or improvements in self-reported salt-lowering behaviours (Table 2). Of the 12 studies whose outcome was salt intake, 10 demonstrated significant reductions ranging from 0.9 g/d to 4.4 g/d [25, 26]. Only two interventions reduced average salt intake by 30% or more post intervention, in line with the WHO global salt target [15, 25]. All four awareness campaigns and four multi-component education interventions significantly improved self-reported behaviours or reduced salt intake. The three studies that demonstrated no significant change in salt intake or behaviour were health education-only programs. Veroff et al. found that mailing a DVD and an accompanying booklet about managing heart failure to participants with heart failure, did not significantly increase the number of participants following a low salt diet [23]. The study by Cotter et al. found the school-based education program about the dangers of high salt intake, with or without a practical gardening component, did not significantly reduce the salt intake of children aged 10–12 years compared to children who did not receive the intervention [31]. In the study by Cappuccio et al., open health education sessions including advice to lower salt intake delivered by community health workers in communal areas in Ghana did not significantly lower 24 h urinary sodium excretion in comparison to the control group who did not receive salt advice [33].

**Table 2:** Intervention characteristics and salt intake or behaviour assessment

Study	Intervention delivery mode	Salt Specific	Theoretical framework/model	Outcome (method of assessment)		
				Salt intake(urine)	Salt intake(dietary survey)	Salt-related behaviours (Q)
Health education						
Ferrara et al. (2012) [36]^a^	Clinic group lectures	N	N		✓ (FFQ)	
Cotter et al. (2013) [31]^a^	School lectures and workshop	Y	N	**=** (24 h)		
Veroff et al. (2012) [23]^a^	Self-led using materials	N	N			**=**
Cappuccio et al. (2006) [33]^a^	Community lectures	Y	N	**=** (24 h)		
Lin et al. (2013) [24], Svetkey (2009) [56]^a^	Clinic group lectures	N	Y	**=** (24 h)	✓ (FFQ)	
Chen et al. (2008) [15]	Workplace lectures	N	N		✓ (Q)	
Kitaoka et al. (2013) [28]	Community lectures	N	N	**=** (spot)	✓ (FFQ)	✓
Ireland et al. (2010) [26]^a^	Community workshop	Y	N	✓ (24 h)	✓ (24 recall)	
Lu et al. (2015) [17]^a^	Community lecture and workshop	N	N		✓ (salt weigh)	
Resnick et al. (2014) [25]	Community lectures	N	Y		✓ (FFQ)	
White et al. (2013) [27]	Community lectures	N	Y		✓ (Q)	
Bogle (2008)	Community workshop	Y	N			✓
Chen et al. (2013) [16]^a^	Community workshop	Y	N	**=** (24 h)	✓ (salt weigh)	✓
Fujii et al. (2009) [29]	Computer program	N	Y			✓
Public awareness campaigns						
Papadakis et al. (2010) [32]^a^	Mass media (province)	Y	Y			✓
Wyness et al. (2012) [20], Millett et al. (2012) [38], He (2014) [54]	Mass media (national)	Y	N			✓
Safefood, Amarach (2006)	Mass media (national)	Y	N			✓
Martins et al. (2009) [30]	Mass media (national)	Y	N			✓
Multi-component education						
Zhang et al. (2014) [18]	Campaign and community lectures	N	N			✓
Khosravi et al. (2012) [24]^a^	Mass media and community lectures	N	N	✓(24 h)		
Drummond (2008)	Campaign and community lecture	Y	N			✓
Surpluss (2008)	Campaign and workplace lecture	Y	N			✓

^a^higher quality studies with more low-risk domains than high-risk domains out of 7; N- No; Y- Yes; ✓- significant effect; = − no significant effect; 24 h - 24 h urine collection; FFQ - food frequency question; Q - questionnaire; 24 recall - 24 h dietary recall; spot - spot urine samples

Nine of 11 salt-specific programs demonstrated significant improvements in a salt-related outcome compared with 10 of 11 broader programs with a component on salt reduction (Table 2). Two of 13 behaviour change interventions delivered in the community did not find a significant decrease in salt intake or improvements in behaviour whereas all interventions delivered nation-wide (4), in workplaces (3) and clinics (2) significantly decreased salt intake or improved salt behaviour. One study of health education delivered to children during school did not significantly change salt intake [31]. All five studies which based their interventions on behaviour change models or theories demonstrated a reduction in salt intake or improved salt-related behaviours [24, 25, 27, 29, 32].

When only considering studies with the higher quality designs or more objective outcome measurements, fewer studies demonstrated a significant reduction in salt intake or improvements in salt behaviour (Table 2). 10 of 24 studies were considered higher quality studies as defined by those with more low risk domains than high risk domains out of seven [16, 17, 23, 24, 26, 31–34, 36]. Of these 10, five studies found the intervention significantly reduced salt intake or improved salt-related behaviours based on the more objective outcome assessment method (when more than one outcome measurement was used) [17, 26, 32, 34, 36]. Three were categorised as health education interventions which involved group lectures to hypertensive patients in the clinic [36] or community [17] setting and education on how to use nutrition labelling to choose low salt foods in healthy adults [26]. The two others were a bilingual mass media campaign encouraging the community to reduce consumption of high salt processed foods [32] and a multi-faceted intervention involving mass media campaigns reinforced with education programs led by health workers in various settings [34]. Two studies used more than one outcome measurement and based on the more objective measurement (24 h urine), there was no significant change in salt intake in both studies however based on the FFQ or salt weighing there was a significant reduction [16, 24]. Studies that used more objective outcome assessment methods were less likely to demonstrate a significant effect. All 9 studies that used dietary surveys or salt weighing to estimate salt intake found a significant reduction. In contrast, based on the gold-standard outcome measure of 24 h urinary sodium excretion [12, 40], only 2 of 6 studies reported significant reductions in salt intake; these were reductions of 1.86 g/d after a multi-component community education intervention in Iran [34] and 0.9 g/d and 1.9 g/d after education on how to choose lower salt foods using the Heart Foundation Tick and the nutrition information panel respectively in Australia [26]. The other 4 studies that did not find a significant reduction based on 24 h urinary sodium excretion include the school education program in children [31], open health education sessions in community centres in Ghana [33], the use of salt-restriction-spoons and health education in Beijing [16] and group education sessions about managing CVD risk reduction led by practitioners trained in behaviour change techniques [24]. Although the last two studies did not demonstrate reductions in salt intake estimated by 24 h urine collection, they found reductions in salt intake based on salt weighing and self-report food frequency questionnaires (FFQs) respectively [16, 24].

## Discussion

This review examined the nature and effectiveness of recent education or awareness-raising interventions that aimed to reduce population salt intake. The majority of studies showed that population-level behaviour change interventions were effective in significantly reducing salt intake (*n* = 10/12) and improving salt-related behaviours (*n* = 11/12). However, when only focusing on higher quality studies, only 5 of 10 studies found a significant reduction in salt intake or improvement in salt lowering behaviours based on the more objective outcome measurement. Similarly, only 2 out of the 6 studies that assessed the impact based on the gold standard measurement of sodium excretion from 24 h urine rather than spot urine, dietary surveys or self-reported behaviours, found a significant reduction. The lack of intervention details in some studies, coupled with the varied nature of interventions and mixed quality of study designs and evaluation measures, meant that results of these programs should be interpreted with caution, and further, that it was difficult to identify specific characteristics contributing to program success or failure.

There was insufficient evidence to show a particular intervention delivery setting was more effective for changing salt-related behaviour. All three interventions delivered in workplaces were successful in reducing salt intake which supports the growing evidence for diet-related programs delivered at worksites [41, 42]. Similarly, there was no evidence to suggest that certain population characteristics influenced the effectiveness of the behaviour change intervention. Behavioural interventions appeared to have similar effects in general populations compared to high risk groups and high income compared to low- and middle-income countries. Among higher quality studies, half of the interventions implemented in the general population (3/6) [26, 32, 34], high risk groups (2/4) [17, 36], high-income countries (3/6) [26, 32, 36] and low and middle income countries (2/4) [17/34] demonstrated a significant reduction in salt intake or improvement in salt behaviour.

This review demonstrated that broader programs which incorporated salt reduction messages were just as likely to be effective as salt-specific interventions for health education and multi-component programs. However awareness campaigns are more likely to be effective if there is a focused and salt-specific message, which is consistent with previous reviews [43]. This review also found some evidence to suggest behaviour change interventions based on theoretical frameworks or models were effective, with all five interventions developed based on theoretical frameworks demonstrating improvements in salt behaviours or a reduction in salt intake. The theoretical frameworks used to develop the interventions include the social ecological model [25], social cognitive theory [24, 25], self-management principles [24, 27], principles of behavioural change [29] and the PRECEDE-PROCEED framework [32]. The added value of using theoretical frameworks to inform interventions is further supported by two recent studies published after the search for this review, which found a multi-component community salt reduction intervention based on the Communication for Behaviour Impact (COMBI) framework significantly reduced salt consumption by 0.8 g/d (*p* < 0.001) as measured by 24 h urine collection in Lithgow and 0.43 g/d based on spot urine samples in Vietnam (*p* = 0.001) [44, 45].

There was no indication that a particular type of population-level behaviour change intervention (health education versus public awareness versus combination of both) was more likely to be effective as all except three studies were effective. However, all of the public awareness campaigns were assessed based on self-reported salt behaviours, not objectively measured salt intake, so the evidence for its effectiveness is weaker. Some lessons are apparent from the studies of health education interventions that showed no change in salt intake or salt-related behaviours. One intervention relied on patients with heart failure to educate themselves using a DVD and materials mailed to their home [23]. The study authors speculated that the impact may have been dampened by not involving clinicians to deliver or discuss the materials provided, which highlights the importance of the delivery mode and involvement of an educator. This is consistent with the study which found a greater proportion of hypertensive participants in the lecture group and the interactive education group consumed less than 6 g/d of salt compared with the self-learning group [17].

The school-based education programs delivered to 10–12 year old children in Portugal found that lectures on the dangers of excess salt intake in combination with gardening of herbs that could be used instead of salt did not significantly reduce salt consumption compared to the group which received no intervention [31]. These findings are in contrast to a recent high quality study in China which showed a school-based education program was effective in reducing salt intake in both children and families of the intervention group as measured by 24 h urinary sodium excretion [46]. Several factors may have contributed to the variation in findings, but a key characteristic of the successful intervention in China was that it incorporated activities that ensured the salt reduction messages were delivered to the children’s families who are often the food preparers. This is consistent with systematic reviews of other childhood diet-related programs which suggest approaches that involve parents and families are often the most effective and sustainable interventions [47].

The high proportion (19/22) of studies which found either a significant reduction in salt intake or improvements in salt-related behaviour should be interpreted with caution. When only considering the 10 higher quality studies, based on each study’s more objective outcome assessment method (e.g. 24 h urine instead of FFQ), only half found a significant reduction in salt intake or improvement in salt-related behaviours [17, 26, 32, 34, 36]. Just over half of all the studies had an uncontrolled study design and therefore alternative explanations for effects observed cannot be disregarded [17, 19–22, 25–27, 37]. Many also used convenience or volunteer samples as opposed to randomly selecting participants, with small sample sizes resulting in non-generalizable results. Furthermore, nearly half of the studies measured effectiveness through self-reported adherence to a low salt diet or salt-lowering behaviours, and these studies were more likely to report improvements in salt behaviour compared with more objective measures of salt intake. This can be explained by previous studies which suggest that the validity of self-reported dietary measures is weakened by social desirability bias, which refers to the tendency to respond questions in a way that is viewed as favourable [48].

In addition, previous work has shown that in countries where the majority of dietary salt comes from processed foods, increases in self-reported salt-lowering behaviours are not always associated with lower salt intakes because high levels of salt across the food supply make it difficult to reduce salt intake even when individuals are attempting salt-lowering behaviours [49]. This also explains why interventions that aim to change people’s behaviour alone have limited effect sizes in some countries, with only two interventions that reported reductions in average salt intake of 30% or more, in line with the WHO salt target. However both were measured using self-report questionnaires which are known to be inaccurate measures of salt intake [50].

As there is uncertainty about the evidence of effectiveness amongst higher quality studies and limited effect size of education or awareness raising interventions in some countries, in line with previous evidence, it is suggested that behaviour change interventions should be implemented in combination with structural interventions or policies that lower the salt content of foods or improve the food environment [51]. However, more research is needed in LMICs where discretionary salt added by consumers remains a major source of salt intake and therefore behaviour change interventions can potentially have a greater impact. Only 6 of 22 included studies were conducted in LMICs despite the fact that 80% of NCD deaths worldwide occur in LMICs and it is rapidly increasing [52, 53].

### Strengths and limitations

A key strength of this review is that all study designs and grey literature were included. Whilst this results in the inclusion of lower quality studies, it also ensures that the breadth of interventions that are being implemented in real-world settings are captured, which adds to external validity and increases the value of the review. Additionally, the assessment of the risk of bias and methodological quality of studies means that the results of the studies can be interpreted in context. However, due to the high level of heterogeneity across all studies, a meta-analysis was considered inappropriate. Several approaches for pooling the results from the higher quality studies were also explored however incompletely reported information, different outcome assessment methods and different analytical approaches limited the ability to perform meaningful analyses. Another limitation of the review was that only studies in English were included.

While outside the scope of this review of behaviour change interventions, other important factors that should be considered are the sustainability of the intervention and its effects, the cost-effectiveness relative to other programs and the varying impact of behaviour change interventions on different socio-economic groups. Our review also only included studies that evaluated behaviour change interventions alone. However, previous studies have suggested behaviour change interventions are most effective when they are implemented with structural policies such as programs to reduce salt content in foods or change the food environment [54, 55]. Behaviour change interventions not only change people’s knowledge, attitudes and behaviour related to salt but it can also generate demand for policy changes and low salt foods, and improve the effectiveness of structural interventions like procurement policies, nutrition labelling schemes and fiscal policies. Future research should consider how these effects are measured to understand the overall benefits of behaviour change interventions. The development of an evaluation framework which incorporates these factors and guides scientifically robust evaluation in real-world settings is required and will provide clearer understanding of characteristics that contribute to the success of population salt reduction programs.

## Conclusion

In summary, this review demonstrates that population-level education and awareness raising interventions can improve salt-related behaviours and/or reduce salt intake. However, the overall quality of the studies is low to moderate. When only considering the 10 higher quality studies, there is mixed evidence with only half demonstrating significant reductions in salt intake or improvements in salt behaviours. Education or awareness campaigns alone are unlikely to be adequate to achieve the WHO target of a 30% reduction in average salt intake, so it should be implemented in combination with structural interventions. The development of an evaluation framework for salt reduction interventions or policies, which encompasses both scientifically rigorous and environmental outcomes in real world settings, would aid much-needed research to better understand and support effective implementation of population salt reduction interventions, particularly in LMICs.

## Abbreviations

COMBI: Communication for behavioural impact
FFQ: Food frequency questionnaire
LMICs: Low- and middle-income countries
NCD: Non-communicable diseases
RCT: Randomized controlled trial
WHO: World Health Organization
